# A combination of genetics and microbiota influences the severity of the obesity phenotype in diet-induced obesity

**DOI:** 10.1038/s41598-020-63340-w

**Published:** 2020-04-09

**Authors:** Margarethe Smoczek, Marius Vital, Dirk Wedekind, Marijana Basic, Nils-Holger Zschemisch, Dietmar H. Pieper, Anja Siebert, Andre Bleich, Manuela Buettner

**Affiliations:** 1 Institute of Laboratory Animal Science, Hannover Medical School, Hannover, Germany; 2 Microbial Interactions and Processes (MINP), Helmholtz Centre for Infection Research (HZI), Braunschweig, Germany; 3 Institute for Medical Microbiology and Hospital Epidemiology, Hannover Medical School, Hannover, Germany

**Keywords:** Genotype, Inflammation, Microbial communities

## Abstract

Obesity has emerged as a major global health problem and is associated with various diseases, such as metabolic syndrome, type 2 diabetes mellitus, and cardiovascular diseases. The inbred C57BL/6 mouse strain is often used for various experimental investigations, such as metabolic research. However, over time, genetically distinguishable C57BL/6 substrains have evolved. The manifestation of genetic alterations has resulted in behavioral and metabolic differences. In this study, a comparison of diet-induced obesity in C57BL/6JHanZtm, C57BL/6NCrl and C57BL/6 J mice revealed several metabolic and immunological differences such as blood glucose level and cytokine expression, respectively, among these C57BL/6 substrains. For example, C57BL/6NCrl mice developed the most pronounced adiposity, whereas C57BL/6 J mice showed the highest impairment in glucose tolerance. Moreover, our results indicated that the immunological phenotype depends on the intestinal microbiota, as the cell subset composition of the colon was similar in obese ex-GF B6NRj^B6JHanZtm^ and obese B6JHanZtm mice. Phenotypic differences between C57BL/6 substrains are caused by a complex combination of genetic and microbial alterations. Therefore, in performing metabolic research, considering substrain-specific characteristics, which can influence the course of study, is important. Moreover, for unbiased comparison of data, the entire strain name should be shared with the scientific community.

## Introduction

The increased intake of dietary lipids combined with physical inactivity in recent decades has resulted in an enormous global health problem. Overweight and obesity are associated with various diseases, such as metabolic syndrome, type 2 diabetes mellitus, and cardiovascular and gastrointestinal diseases^1^. Overweight and obesity are defined as abnormal or excessive adipose tissue accumulation that may impair health. According to the WHO, overweight (BMI > 25 kg/m^2^) and obesity (BMI > 30 kg/m^2^) affect 1.9 billion people worldwide^2^. In addition to environmental factors, genetic susceptibility and altered microbial diversity were identified to increase the risk of obesity^3,4^.

Several animal models have been established to analyze these factors separately and in a standardized manner^5^. Diet-induced obesity (DIO) models are often utilized during metabolic research, as they resemble human obesity^6^. Diets rich in fat primarily induce adiposity as well as insulin resistance, impaired glucose tolerance and hyperlipidemia^5^. The inbred C57BL/6 (B6) mouse strain is often used for various experimental investigations, including for metabolic research. Over the years, several genetically distinguishable B6 substrains have evolved, including the substrains C57BL/6 J (B6J) and C57BL/6 N (B6N)^7^. The phenotypic manifestation of genetic alterations has resulted in behavioral and metabolic differences among these substrains^8–10^. Moreover, differences in the DIO response were detected. One of the most commonly described genetic differences between B6 substrains is a mutation within the nicotinamide nucleotide transhydrogenase (*Nnt*) gene^11^. This mutation has been linked to impaired glucose metabolism and insulin secretion. Several other genetic differences between the B6 substrains were identified using single nucleotide polymorphism (SNP) genotyping.

Additionally, host genetics and nutrition influence the intestinal microbiota^12,13^. In both humans and mice, obesity has been associated with reduced diversity and characteristic changes in the microbiota^14,15^.

Furthermore, obesity is associated with low-grade chronic inflammation characterized by an increased accumulation of immune cells and proinflammatory cytokines such as interleukin (IL)-6 and tumor necrosis factor alpha (TNFα)^16,17^. Elevated levels of the inflammatory mediator leptin are distinctive during obesity^18^. This adipokine promotes the proliferation of human blood mononuclear cells, induces the secretion of proinflammatory cytokines by macrophages and increases CD4^+^ T cell proliferation in the Th1 phenotype. Immunological differences between B6 substrains have been described previously. B6N mice exhibited a more activated and proinflammatory phenotype than B6J mice^10,19,20^. However, immunological differences among the substrains under conditions of obesity need to be further analyzed.

In this study, a comparison of DIO in C57BL/6JHanZtm (B6JHanZtm), C57BL/6NCrl (B6NCrl) and C57BL/6J (B6J) mice revealed several genetic, metabolic, microbial and immunological differences between the B6 substrains.

## Results

### DIO results in strain-dependent metabolic differences

**Figure 1:**
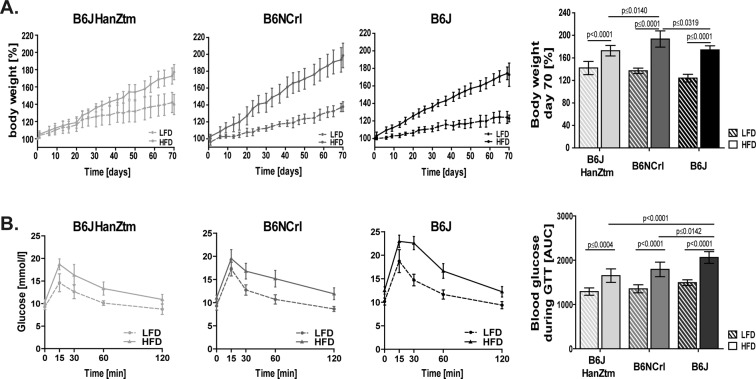
Obesity-induced differences in body weight and glucose tolerance in B6 substrains. Body weight and glucose tolerance were analyzed after 10 weeks of feeding a LFD or HFD. Two independent experiments were performed. (**A**) Body weight in % was measured twice per week and calculated at the initiation of LFD or HFD feeding (n = 9–11; mean ± 95%Cl). Body weight at day 70 (n = 9–11; mean ± 95%Cl, two-way ANOVA with Sidak’s multiple comparisons test). (**B**) During the GTT, blood glucose levels were determined before and at 15, 30, 60 and 120 min after administration of a glucose solution. For analysis, concentration-time curves were created (n = 9–11; mean ± 95%Cl). The AUC was calculated from the concentration-time curves (n = 9–11; mean ± 95%Cl, two-way ANOVA with Sidak’s multiple comparisons test).

Our analysis revealed that all mice continuously gained body weight during the study. However, high fat diet (HFD) feeding induced obesity, as HFD-fed mice showed a 1.2- to 1.4-fold higher body weight increase than low-fat diet (LFD)-fed mice (Fig. 1A). The most rapid body weight gain was detected in obese B6NCrl mice, whereas obese B6J and B6JHanZtm mice showed a less pronounced body weight gain. Body weight gain is associated with glucose intolerance. Blood glucose levels during the oral glucose tolerance test (GTT) were similar in lean mice but increased in obese mice (Fig. 1B). Moreover, the oral GTT revealed increased glucose intolerance in obese B6J mice compared to obese B6JHanZtm and B6NCrl mice.

**Figure 2:**
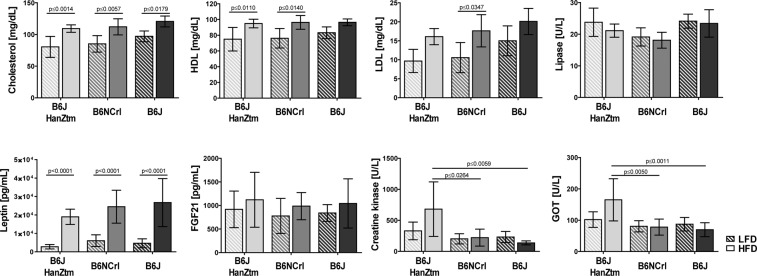
Serum parameters in lean and obese mice. Concentrations of cholesterol, HDL, LDL, lipase, CK and GOT were measured in the serum of LFD- and HFD-fed mice from each B6 substrain (n = 8–11; mean ± 95%Cl two-way ANOVA with Sidak’s multiple comparisons test). Concentrations of leptin and FGF21 were measured in the serum using a magnetic bead-based multiplex assay (n = 5–11; mean ± 95%Cl, two-way ANOVA with Sidak’s multiple comparisons test). Two independent experiments were performed.

The concentrations of different serum parameters were analyzed following dissection. Levels of cholesterol, high-density lipoprotein (HDL), low-density lipoprotein (LDL) and leptin were increased in obese mice of all B6 substrains (Fig. 2). The analysis revealed strain-dependent differences. Concentrations of cholesterol and LDL tended to be increased in B6J mice compared to B6JHanZtm and B6NCrl mice. (Cholesterol: two-way ANOVA, diet: p < 0.0001, F(1, 53) = 43.61; strain: p ≤ 0.0169 F(2, 53) = 4.408. LDL: two-way ANOVA, diet: p < 0.0001, F(1, 54) = 23.44; strain: p ≤ 0.0124 F(2, 54) = 4.766). Furthermore, increased creatine kinase (CK) and glutamic oxaloacetic transaminase (GOT) levels were detected only in obese B6JHanZtm mice, whereas lipase levels tended to be reduced in B6NCrl mice (CK: two-way ANOVA, strain: p ≤ 0.0049 F(2, 50) = 5.936. GOT: two-way ANOVA, interaction: p < 0.0426, F(2, 52) = 3.356; strain: p ≤ 0.0018 F(2, 52) = 7.181. Lipase: two-way ANOVA, strain: p ≤ 0.0020 F(2, 50) = 7.037) (Fig. 2).

### Genetic differences among the B6 substrains

**Figure 3:**
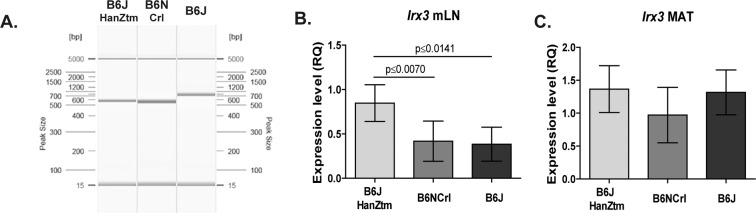
Genetic alterations among the substrains. Obesity-associated genes (*Nnt* and *Irx3*) were analyzed. (**A**) Representative results of capillary electrophoresis analysis after amplification of the *Nnt* wild-type allele (579 bp) and *Nnt* mutant allele (743 bp) from DNA isolated from B6JHanZtm, B6NCrl and B6J mice by using a three primer, two allele-specific PCR assay. (**B**+**C**) Relative gene expression of *Irx3* in the mLN (n = 6–11; mean ± 95%Cl, one-way ANOVA with Tukey’s multiple comparisons test) and MAT (n = 10; mean ± 95%Cl) was measured by qPCR and normalized to a reference sample set to 1.

SNP genotyping was performed using 39 SNPs that differentiate several B6 substrains. Our analysis revealed that all three substrains shared the same allele at only 3 locations, whereas 35 loci differed between B6NCrl and B6J mice (Table 1). Allelic variation was observed at 17 locations between B6JHanZtm and B6J mice and at 19 locations between B6JHanZtm and B6NCrl mice. Some of these SNP loci were linked to genes, such as *Wsb1*, *Snap29* and *Aplp2*, suggesting that they play a role in obesity development^21,22^. Furthermore, the *Nnt* mutation in B6J mice has been associated with glucose intolerance^11^. This *Nnt* mutation was detected solely in B6J mice. In B6NCrl and B6JHanZtm mice, the wild-type *Nnt* allele was verified (Fig. 3A).

**Table 1:** SNP analysis of B6JHanZtm, B6NCrl and B6J mice.

SNP	Chr	Position	Linked genes	B6JHanZtm	B6NCrl	B6J	Obesity associated Ref
08-015199792-M	8	15199792		C:C	C:C	T:T	
11-004367508-M	11	4367508		G:G	A:A	G:G	
13-041017317-M	13	41017317		T:T	C:C	T:T	
15-057561875-M	15	57561875		A:A	G:G	A:A	
19-049914266-M	19	49914266		T:T	T:T	G:G	
rs13459107	9	28298786		G:G	G:G	A:A	
rs13459122	10	80795365		T:T	A:A	T:T	
rs13459145				G:G	G:G	A:A	
rs13475814	1	36879483	Tmem131	T:T	T:T	T:T	
rs13476801	2	138480020		C:C	C:C	T:T	
rs13476956	3	5370727	Zfhx4	C:C	T:T	C:C	^55^
rs13477019	3	23824920	Naaladl2	A:A	A:A	T:T	^56,57^
rs13477132	3	58109942		C:C	C:C	G:G	
rs13477622	4	28322410		T:T	C:C	T:T	
rs13477746	4	65944235	Astn2	T:T	C:C	T:T	^58,59^
rs13477863	4	98297639		A:A	A:A	A:A	
rs13478736	6	46421423	Cntnap2	T:T	T:T	T:T	
rs13478783	6	60591379		A:A	G:G	A:A	
rs13478995	6	117470880		G:G	G:G	C:C	
rs13479233	7	47816324		G:G	G:G	T:T	
rs13479522	7	129035694		A:A	G:G	A:A	
rs13479733	8	43875316		G:G	G:G	A:A	
rs13480122	9	31156626	Aplp2	T:T	C:C	T:T	^21^
rs13480619	10	57752462		T:T	C:C	T:T	
rs13480759	10	109378627		C:C	T:T	C:C	
rs13480829	10	129350405	Olfr782	A:A	A:A	G:G	
rs13481014	11	48117382		T:T	C:C	T:T	
rs13481117	11	79252230	Wsb1	G:G	T:T	G:G	^22^
rs13481439	12	48965551		A:A	G:G	A:A	
rs13481573	12	86909001		G:G	A:A	G:G	
rs13481634	12	106833655		C:C	C:C	A:A	
rs13481676	13	6304055		A:A	G:G	A:A	
rs13481734	13	27037150		G:G	G:G	A:A	
rs13483055	17	60319945		C:C	C:C	T:T	
rs13483296	18	35206506		T:T	T:T	A:A	
rs13483369	18	54614841	9330117O12Rik	C:C	C:C	A:A	
rs13483883	15	7167980		A:A	A:A	G:G	
rs31233932	14	124108797	Fgf14	C:C	T:T	C:C	^60,61^
rs4165065	16	17412079	Snap29	T:T	C:C	T:T	^21^

Positions of the SNPs are in accordance with dbSNP release 150.

Another candidate gene for obesity is *Irx3* (Iroquois-related homeobox 3)^23^. Neither genetic variations in the *Irx3* gene sequence nor differences in the isoform transcripts were detected between the substrains (Suppl. Fig. 1). However, differences were observed in *Irx3* gene expression in DIO (Fig. 3B,C). *Irx3* expression in the mesenteric lymph nodes (mLN) was increased in B6JHanZtm mice compared to that in B6NCrl and B6J mice (Fig. 3B) and tended to be increased in MAT of both B6J and B6JHanZtm mice (Fig. 3C).

### DIO results in strain-dependent immune activation

**Figure 4:**
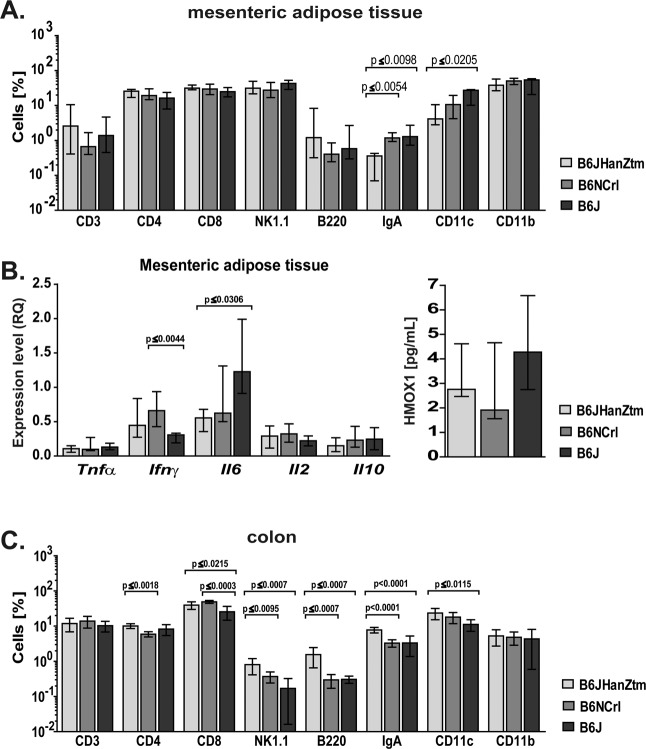
Immunological differences in the MAT and colon of obese mice. Obesity-induced differences in cell subset composition of MAT and colon as well as cytokine expression and HMOX1 levels in the MAT were detected between the substrains. (**A**) Surface staining of total cell populations of the MAT (n = 5–11; median ± IQR[25–75], Kruskal-Wallis test with Dunn’s multiple comparisons test) from obese mice was performed and analyzed by flow cytometry. CD3^+^ cells, B220^+^ cells, IgA^+^ cells and MHCII^+^ cells were gated from the leukocyte gate of the MAT. NK1.1^+^ cells and CD4^+^ and CD8^+^ cells were gated from CD3^+^ cells. CD11c^+^ cells and CD11b^+^ cells were gated from MHCII^+^ cells. Amounts are presented on a logarithmic scale. (**B**) Relative gene expression of cytokines and HMOX1 levels in the MAT of obese mice were measured by qPCR and normalized to a reference sample set to 1 (n = 4–8; IQR[25–75], Kruskal-Wallis test with Dunn’s multiple comparisons test) or ELISA (n = 5–6; median ± IQR[25–75]), respectively. (**C**) Flow cytometry staining of the total cell population from the colon (n = 5–6; mean ± 95%Cl, one-way ANOVA with Tukey’s multiple comparisons test) of obese mice was performed and analyzed as described above. Amounts are presented on a logarithmic scale.

Obesity is associated with low-grade chronic inflammation. In our study, immunological differences were detected in the cell subset composition of the MAT and colon among the obese mice of the B6 substrains (Fig. 4).

In the MAT, the numbers of MHCII^+^CD11c^+^ and IgA^+^ cells in B6J mice were higher than those in B6JHanZtm mice. IgA^+^ cells were also increased in B6NCrl mice compared to those in B6JHanZtm mice (Fig. 4A). *Ifnγ* expression levels were higher in B6JHanZtm and B6NCrl mice, whereas *Il6* levels were increased in B6J mice (Fig. 4B). Additionally, HMOX1 concentrations tended to be increased in the MAT of B6J mice (Fig. 4B). Several differences were observed in the cell subset composition of the colon among the B6 substrains (Fig. 4C). CD8^+^ T cells were increased in the B6NCrl substrain compared to the other B6 substrains. Furthermore, higher numbers of NK1.1^+^ T cells, B220^+^ cells, IgA^+^ cells and CD11c^+^ cells were detected in B6JHanZtm mice (Fig. 4C).

### Strain- and diet-dependent differences are associated with intestinal microbiota composition

**Figure 5:**
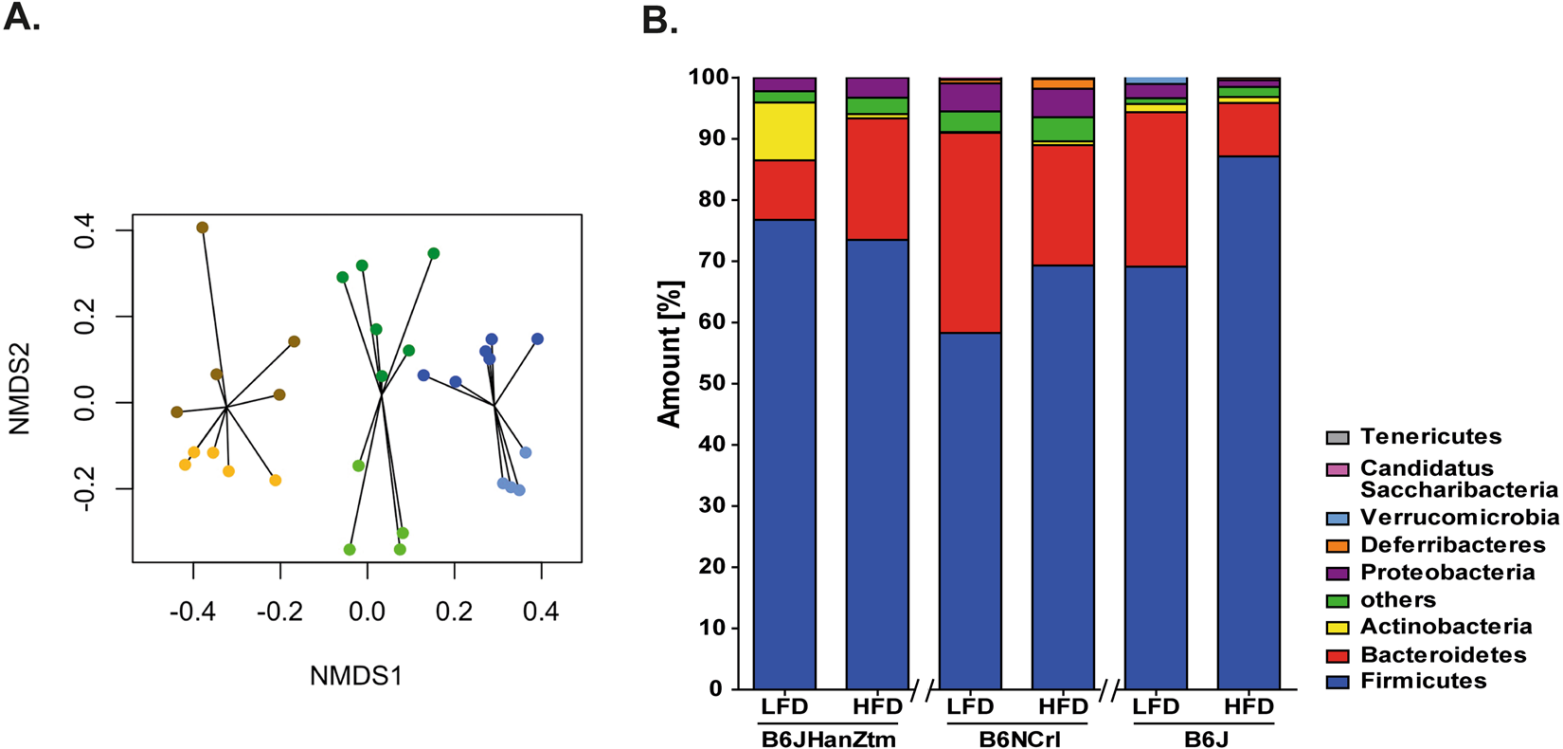
Obesity-induced alterations of the microbiota in a substrain-dependent manner. Microbial communities in LFD- and HFD-fed mice of each B6 substrain were analyzed. (**A**) NMDS diagram of microbial communities in the B6 substrains. Each dot represents the microbiome composition of a single animal. Light colors: LFD. Dark colors: HFD. Blue: B6J. Green: B6JHanZtm. Yellow: B6NCrl. (**B**) Frequencies of the most common phyla in the intestinal content from LFD- and HFD-fed mice of each B6 substrain.

Microbiome analysis was performed on cecal contents from lean and obese mice of each substrain to detect differences in the microbial community. Nonmetric multidimensional scaling (NMDS) analysis revealed strain- and diet-dependent clusters of microbiota (Fig. 5A). In line with this finding, differences in the community composition based on both factors, strain and diet, were observed (permutational ANOVA; adonis (formula = TogNCTH ~ Diet + Strain, permutations = 10000), diet: p < 0.01, strain: p < 0.01). *Firmicutes* was the most frequent phylum, accounting for approximately 60–85% of the total bacterial sequences in all B6 substrains (Fig. 5B). Other common phyla were *Bacteroidetes* (10–30%), *Proteobacteria* (up to 5%) and *Actinobacteria* (<10%) in lean B6JHanZtm mice only (Fig. 5B). Additionally, a reduced abundance of *Bacteroidetes* and an increased frequency of *Firmicutes* were observed in obese B6J and B6NCrl mice. In B6JHanZtm mice, *Firmicutes* abundance was similar between lean and obese mice, whereas those of *Bacteroidetes* were increased in obese mice.

### Microbiome transfer

**Figure 6:**
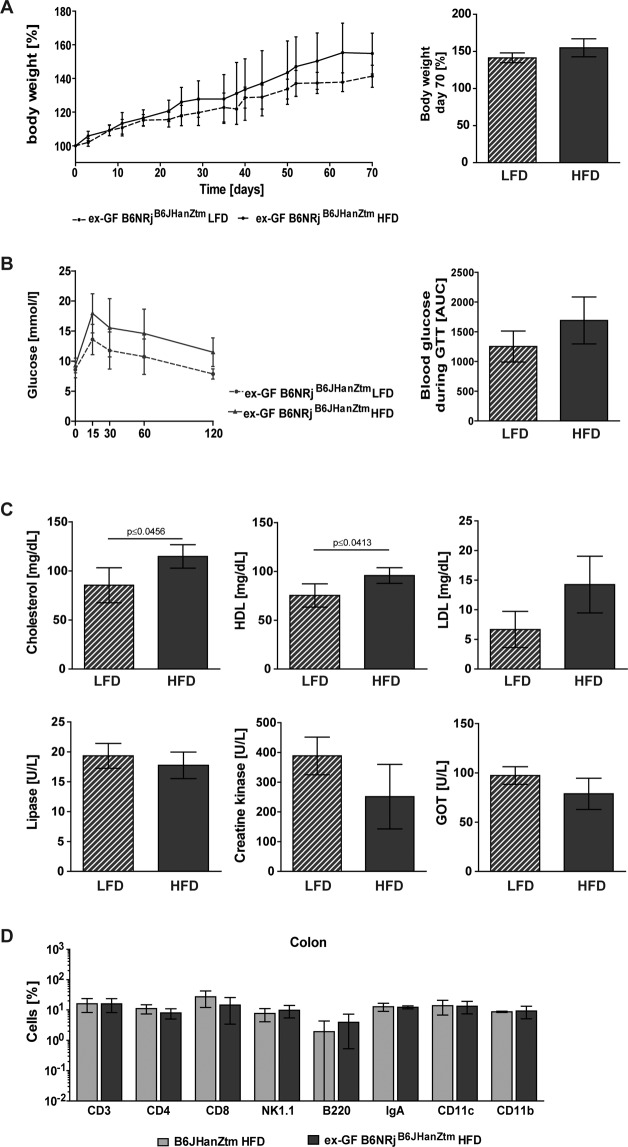
Microbiota and genetics are involved in obesity-induced parameters. GF C57BL/6NRj mice were cohoused with B6JHanZtm mice over a period of 4 weeks (GF C57BL/6NRj mice referred to as ex-GF B6NRj^B6JHanZtm^). Subsequently, ex-GF B6NRj^B6JHanZtm^ mice were fed a LFD or HFD for 10 weeks. Two independent experiments were performed. (**A**) Body weight in % was measured twice per week and calculated at the initiation of LFD or HFD feeding and at day 70 (n = 3–4; mean ± SD). (**B**) During the GTT, blood glucose levels were determined before and at 15, 30, 60 and 120 min after administration of a glucose solution. For analysis, concentration-time curves were created, and the AUC was calculated (n = 3–4; mean ± SD). **C:** Concentrations of cholesterol, HDL, LDL, lipase, CK and GOT were measured in the serum of LFD- and HFD-fed ex-GF B6NRj^B6JHanZtm^ mice (n = 3–4; mean ± SD, unpaired t test). **D:** Surface staining of the total cell population of the colon from HFD-fed ex-GF B6NRj^B6JHanZtm^ mice and their microbiota donor mice (obese B6JHanZtm) was performed and analyzed by flow cytometry. CD3^+^ cells, B220^+^ cells, IgA^+^ cells and MHCII^+^ cells were gated from the leukocyte gate of the colon. NK1.1^+^ cells and CD4^+^ and CD8^+^ cells were gated from CD3^+^ cells. CD11c^+^ cells and CD11b^+^ cells were gated from MHCII^+^ cells. Amounts are presented on a logarithmic scale (n = 2–4; mean ± SD).

Microbiota transfer experiments were performed to investigate wether transplantation leads to a transfer of phenotype as well. HFD-fed ex-GF B6NRj^B6JHanZtm^ mice showed a less pronounced body weight increase (Fig. 6A) compared to obese B6JHanZtm mice (Fig. 1A). Furthermore, HFD-fed ex-GF B6NRj^B6JHanZtm^ mice developed glucose intolerance and hyperlipidemia comparable to those observed in obese B6JHanZtm mice (Figs. 1 and 6B,C). Serum levels of lipase, CK and GOT showed no differences between lean and obese ex-GF B6NRj^B6JHanZtm^ mice (Fig. 6C). Analysis of the cell subsets in the colon revealed a similar composition in obese B6JHanZtm and ex-GF B6NRj^B6JHanZtm^ mice (Fig. 6D).

**Figure 7:**
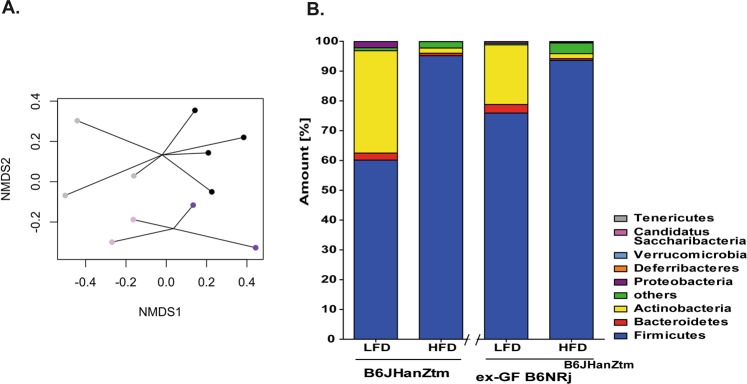
Microbiota transfer to germfree mice resulted in similar bacterial community frequencies. Microbial communities in LFD- and HFD-fed B6JHanZtm and ex-GF B6NRj^B6JHanZtm^ mice were analyzed. Frequencies of the most common phyla and NMDS diagram of microbial communities. Each dot represents the microbiome composition of a single animal. Light colors: LFD. Dark colors: HFD. Violet: B6JHanZtm. Gray: ex-GF B6NRj^B6JHanZtm^.

The intestinal microbiota of lean and obese ex-GF B6NRj^B6JHanZtm^ mice were analyzed and compared with those of B6JHanZtm mice (Fig. 7), and NMDS revealed diet- and strain-dependent clusters of microbial communities (Fig. 7A). Again, *Firmicutes* was the most abundant phylum in ex-GF B6NRj^B6JHanZtm^ mice (75–95%). Similar to lean B6JHanZtm mice, a high abundance of *Actinobacteria* was observed only in lean ex-GF B6NRj^B6JHanZtm^ mice. However, the frequency of *Firmicutes* was increased, whereas the frequency of *Actinobacteria* was reduced in lean ex-GF B6NRj^B6JHanZtm^ mice compared to those in lean B6JHanZtm mice (Fig. 7B). Thus, differences in the microbiota of these mice might be mediated by host genetics.

## Discussion

Obesity has emerged as a major global health problem and is associated with various diseases such as metabolic syndrome, type 2 diabetes mellitus, and cardiovascular and gastrointestinal diseases^1^. In addition to environmental factors, such as an increased intake of dietary lipids, genetic susceptibility and microbial diversity have been identified as increasing the risk of obesity^3,4,24^.

The B6 inbred mouse strain is often used for various experimental investigations, such as metabolic research. However, over the years, genetic drift has led to B6 substrains^7,25^. The phenotypic manifestation of genetic alterations has resulted in behavioral and metabolic differences^8,10^. Our comparison of DIO in B6JHanZtm, B6NCrl and B6J mice revealed several metabolic, genetic, microbiological and immunological differences among these B6 substrains. Because standard thresholds (such as BMI) are not available in animals, a 10–25% higher body weight than control-fed mice was defined as moderate obesity and a 40% higher body weight as severe obesity^5^. In this study, HFD-fed B6J and B6NCrl mice showed a more than 40% increase in body weight compared to lean mice and were characterized as severely obese. In contrast, B6JHanZtm mice were characterized as moderately obese.

Various differences in behavior, phenotype and genetics were described previously between B6N and B6J mice^8,21,26^. A well-known genetic difference between B6N and B6J mice is the *Nnt* mutation in B6J mice^11^. The *Nnt* gene encodes a mitochondrial enzyme that is involved in NADP^+^ reduction to NADPH^27^. The mutation leads to reduced NNT production and has been linked to glucose intolerance and beta cell function^28,29^. Differences in glucose tolerance between B6N and B6J mice have been described previously^11^, but similarities in glucose tolerance were also observed^30,31^. In our study, the *Nnt* mutation was detected only in B6J mice, whereas the wild-type *Nnt* allele was determined in B6NCrl and B6JHanZtm mice. This spontaneous mutation within the *Nnt* gene arose between 1976 and 1984^32^. As B6N and B6JHanZtm mice diverged from B6J mice before 1976, they inherited the wild-type *Nnt* allele^7,26^. Accordingly, obese B6J mice have shown the highest impairment in glucose tolerance. Thus, our results strengthened the association between the *Nnt* mutation and glucose intolerance.

In addition to the *Nnt* mutation, further genetic differences between B6N and B6J mice have been published^7,33^. Backcrossing experiments between B6N and B6J mice revealed 4 SNPs (rs13481014, rs13480122, rs13478783 and rs4165065) associated with increased body weight^7,21^. In our genetic analysis, these four SNPs were also found to differ between B6NCrl and B6J/B6JHanZtm mice. Therefore, these SNPs might be involved in the more pronounced body weight gain of B6NCrl mice compared to that of B6J and B6JHanZtm mice. The genetic profile of B6JHanZtm mice shared similarities with that of both B6NCrl and B6J mice, but obesity progression was less severe than in B6J and B6NCrl mice. Thus, our results indicated that differences in body weight gain depend on other genetic differences. Genes associated with human obesity are *FTO* and *IRX3*^34,35^. Experimental depletion of both genes induced a reduction in body weight compared to wild-type mice^36,37^. FTO and IRX3 were detected in various organs, such as brain and adipose tissue^37,38^. Analysis of *Irx3* expression revealed similar expression in the MAT of obese mice. *Irx3* expression was substantially increased in the mLN of obese B6JHanZtm mice compared to that in obese mice of both other strains. Thus, *Irx3* does not seem to be the cause of the differences in body weight gain, but the role of *Irx3* expression in mLN needs to be further analyzed.

Various studies illustrated a strong influence of microbiota on obesity^4,39–41^. Germfree mice are largely protected against obesity^42,43^. However, the transplantation of uncultured feces from obese donors into germfree mice leads to obesity, whereas mice transplanted with feces of lean donors do not develop obesity^4,44^. Obesity in both humans and mice has been associated with reduced diversity and characteristic changes in the gut microbiota^14,15^. A common change in the microbiota during obesity is an altered *Bacteroidetes*/*Firmicutes* ratio. In our study clusters of microbial communities that were strain- and diet-dependent were detected during the NMDS analysis and confirmed by the statistical analysis. The analysis of the microbiota revealed reduced abundance of *Bacteroidetes* and increased frequency of *Firmicutes* in obese B6NCrl and B6J mice. In contrast, *Bacteroidetes* were additionally increased in obese B6JHanZtm mice and *Firmicutes* abundance was similar between lean and obese B6JHanZtm mice in this experimental setup. However, the frequency of *Firmicutes* increased in HFD donor mice during the GF co-housing experiment. One reason could be an altered microbiota in B6JHanZtm mice on the species level depending on the age of the mice which seemed to have an impact on the whole bacteria composition. This is in line with others who presented age-dependent microbiota changes^45–47^. In addition, high levels of *Actinobacteria* (up to 45%) were found only in lean B6JHanZtm mice. The microbial alterations among the B6 substrains possibly contributed to the differences in obesity.

During our analysis of the influence of strain-specific microbiota, HFD-fed ex-GF B6NRj^B6JHanZtm^ mice showed a less pronounced body weight increase. However, HFD-fed ex-GF B6NRj^B6JHanZtm^ mice developed glucose intolerance and hyperlipidemia similar to those observed in obese B6JHanZtm mice. We detected differences in the microbial composition between B6JHanZtm and ex-GF B6NRj^B6JHanZtm^ mice. Diet- and strain-dependent clusters of microbial communities were observed during NMDS. Analysis of the frequencies revealed similar microbial compositions in obese B6JHanZtm and obese ex-GF B6NRj^B6JHanZtm^. An increased frequency of *Firmicutes* and reduced frequency of *Actinobacteria* were observed in lean ex-GF B6NRj^B6JHanZtm^ mice compared to those in lean B6JHanZtm mice. Such differences in the microbiota in these mice might be mediated by host genetics. Previous studies have reported that host genetics have a partial influence on the microbiota^13,48,49^. Thus, the severity of obesity- and obesity-related alterations in metabolism is likely defined by a specific combination of microbiota, host genetics and environment. Furthermore, host genetics and the microbiota influence the immunological phenotype. Immunological differences among mouse strains are well documented, but differences were also observed among substrains^50^. Several studies reported an increased proinflammatory response in B6N mice compared to that in B6J mice^10,20^. Macrophages from B6N mice displayed a more activated M1 phenotype and produced more NO than those from B6J mice in a tumor model^19^. Obesity is often described as a chronic state of low-grade inflammation^16,17,48^. In our study, immunological differences between the substrains were also detected during obesity. However, most differences were observed between B6JHanZtm mice and both B6NCrl and B6J mice. For example, MHCII^+^CD11c^+^ and IgA^+^ cells were reduced in the MAT of B6JHanZtm mice, whereas NK1.1^+^ T cells, B220^+^ cells, IgA^+^ cells and CD11c^+^ cells were increased in the colon of these mice compared to those of both other B6 substrains. Comparison of B6NCrl and B6J mice revealed a strikingly similar immunological phenotype during obesity. Moreover, the cell subset composition of the colon was similar between obese ex-GF B6N^B6JHanZtm^ and obese B6JHanZtm mice. Consequently, our results indicated that the immunological phenotype is additionally transferred during cohousing experiments and largely depends on the transferred microbiota.

Finally, our results corroborated previously published data demonstrating several genetic and phenotypic differences between B6 substrains during obesity. These differences are caused by a combination of genetic and microbial alterations. Therefore, in performing metabolic research, considering substrain-specific characteristics, which can influence the course of study, is important. Moreover, for unbiased comparisons of data, the entire strain name should be shared with the scientific community.

## Methods

### Mice

Male mice at a body weight of 18–25 g were used in this study. C57BL/6 JHanZtm (B6JHanZtm) and germfree (GF) C57BL/6NRj (B6NRj) mice were obtained from the Central Animal Facility of the Hannover Medical School (MHH; Hannover, Germany). C57BL/6NCrl (B6NCrl) and C57BL/6J (B6J) mice were purchased from Charles River (Sulzfeld, Germany).

### Ethical statement

This study was conducted in accordance with German animal protection laws and with the European Directive 2010/63/EU. All experiments were approved by the Local Institutional Animal Care and Research Advisory committee and permitted by the Lower Saxony State Office for Consumer Protection and Food Safety (LAVES; file number: 13/1174).

### Feeding and oral glucose tolerance test

Mice of each substrain were fed a HFD (D12492, Research Diets, New Brunswick, USA) containing 60% kcal fat or a low-fat diet (LFD; D12450J, Research Diets, New Brunswick, USA) that contained 10% kcal fat ad libitum for 10 weeks.

To investigate the influence of strain-specific microbiota, four-weeks-old GF B6NRj mice were cohoused with B6JHanZtm mice over a period of 4 weeks in gnotocages^51^ for microbiota transfer (GF B6NRj mice will henceforth be referred to as ex-GF B6NRj^B6JHanZtm^). Subsequently, ex-GF B6NRj^B6JHanZtm^ mice were fed a LFD or HFD for 10 weeks.

During the feeding period, body weight was determined twice per week, and an oral GTT was performed at the end of the study. For the GTT, mice were fasted for 6 h and then administered a glucose solution (2 g/kg) by oral gavage. Blood glucose levels were determined at different time points (0, 15, 30, 60 and 120 min) using a glucose meter (Contour XT, Bayer, Leverkusen, Germany).

### Serum analysis

After 70 days of feeding, mice were sacrificed by CO2 inhalation followed by exsanguination through cardiac puncture. The obtained serum was diluted 1:2 with saline solution, and the concentrations of cholesterol, HDL, LDL, lipase, CK and GOT were determined at the Institute for Clinical Chemistry of the MHH.

The levels of TNFα, leptin, FGF-21, MCP-1, IFNγ and IL-6 in diluted serum were measured using a Magnetic Luminex^®^ Screening Assay (R&D Systems, Wiesbaden, Germany). The assay was performed according to the manufacturer’s instructions, and concentrations were determined by parallel standard curves for each parameter.

### SNP genotyping

DNA was isolated from tail biopsies by using a MasterPure™ complete DNA Purification Kit (Lucigen, Middleton, USA) in accordance with the manufacturer’s instructions. SNP genotyping was carried out by LGC Genomics (Hoddesdon, UK).

### Allele specific PCR for the Nnt gene

Mutations in the *Nnt* gene were assessed using a three primer, two allele PCR assay as previously described^11^. The thermocycling conditions wereas follows: (i) an initial denaturation step of 300 s at 95 °C; (ii) 30 cycles of 45 s at 95 °C, 30 s at 58 °C (annealing temperature) and 45 s at 72 °C; and (iii) a final elongation step of 600 s at 72 °C. After amplification, the size of the PCR product was examined by capillary electrophoresis (QIAxcel advanced, Qiagen, Hilden, Germany).

### Irx3 gene evaluation

The Irx3 alleles of the mouse strains B6JHanZtm, B6NCrl and B6J were amplified with Q5 high-fidelity Taq polymerase (New England Biolabs, Ipswich, MA, USA) using the primer pair 5'-GACGACAGGAGGAGAGTGTAAACTAG-3' and 5'-GGCAGACCTGCCGGTTATAGTCAAAA-3', producing a fragment that covered 3136 bp of the B6J allele (ACCESSION No: NC_000074.6). The thermocycling conditions were as follows: (i) an initial denaturation step of 30 s at 94 °C; (ii) 35 cycles of 30 s at 98 °C, 30 s at 60 °C (annealing temperature) and 210 s at 72 °C; and (iii) a final elongation step of 600 s at 72 °C. The PCR products were directly cloned into the TOPO TA-cloning vector pSC-A amp/kan (Agilent, Waldbronn, Germany). Two independent clones from each strain were sequenced (Eurofins Genomics, Ebersberg, Germany) with vector-specific T3 and T7 oligonucleotides and with five Irx3-specific primers (Irx3_1: 5′-TCTGGGTCCCTATCCAATGTG-3′, Irx3_2: 5′-AGGAGAACAAGATGACGTGG-3′, Irx3_3: 5′-AGAAGCCCAAGATCTGGTCA-3′, IRX3_4: 5′-TCCTACAGATCGCTGTAGTG-3′, Irx3_5: 5′-CTCTGGTCTTATCAGCTCT-3'). Sequence analysis and alignment were performed with ApE-A Plasmid Editor v2.0.47 software.

### Irx3 isoform analysis

RNA was isolated from mesenteric lymph nodes (mLNs) and cDNA was synthesized as described above except for the lysis procedure. To distinguish between isoform variant 1 (ACCESSION No: NM_008393.3) and isoform variant 2 (ACCESSION No: NM_001253822.1) of IRX3 mRNA in the mice, which have a sequence difference of 14 base pairs, PCR with the primer pair 5′-AGCCGGAGAGTGGAACAG-3′ and 5′-CCACTTCCAAGGCTCTACAG-3′ was performed, with expected product sizes of 56 bp and 42 bp, respectively. The obtained PCR products were analyzed by 10% bisacrylamide-TBE gel electrophoresis using the OneTaq Hot Start 2x Master Mix (New England Biolabs, Ipswich, MA, USA). The thermocycling conditions were as follows: (i) an initial denaturation step of 30 s at 94 °C; (ii) 30 cycles of 10 s at 98 °C, 10 s at 52 °C (annealing temperature) and 10 s at 68 °C; and (iii) a final elongation step of 30 s at 68 °C.

### Quantitative real-time PCR (RT-qPCR)

Before RNA isolation, mesenteric adipose tissue (MAT) was lysed at 37 °C for 5 min in RLT (RNeasy Mini Kit, Qiagen, Hilden, Germany), homogenized with a sonicator and then centrifuged for 5 min at 2500 × *g*. The aqueous phase was used for further analysis. The following RNA isolation steps were performed as described previously^52^. cDNA was synthesized by using a QuantiTect Reverse Transcription Kit (Qiagen, Hilden, Germany) according to the manufacturer’s protocol. The cDNA obtained for quantitative real-time PCR was performed using TaqMan^®^ Fast Advanced Master Mix and TaqMan^®^ Gene Expression Assays for *Irx3* (Mm00500463_m1), *Tnfα* (Mm00443258_m1), *Ifnγ* (Mm01168134_m1), *Il6* (Mm00446190_m1), *Il2* (Mm00434256_m1), and *Il10* (Mm01288386_m1); *β-actin* (Mm00607939_s1) was used as an endogenous control (all acquired from Thermo Fisher Scientific, Waltham, USA). Gene expression was determined in a StepOnePlus™ Real-Time PCR System (Applied Biosystems, Weiterstadt, Germany). The thermocycling conditions for the TaqMan® chemistry were as followed: (i) an incubation step of 120 s at 50 °C; (ii) a polymerase activation step of 20 s at 95 °C; and (iii) 40 cycles of 1 s at 95 °C and 20 s at 60 °C (annealing and elongation step). All reactions were run in triplicate. Relative gene expression was calculated in relation to a reference sample using the 2^−ΔΔCt^ method.

### Flow cytometry

Cell suspensions were prepared from MAT and colon tissue. MAT was digested at 37 °C for 20 min with 0.75 mg/mL collagenase (from *Clostridium histolyticum*, Type VIII, Sigma Aldrich, Steinheim, Germany) in Hanks’ Salt Solution (HSS; Biochrom, Berlin, Germany). Colon tissue, devoid of attached mesentery and adipose tissues, was rinsed carefully with PBS. Thereafter, the colon tissue was cut longitudinally and incubated in buffer I (3.5% FCS, 100 mM DTT in HSS) at 37 °C for 20 min. Subsequently, the colon tissue was removed and treated twice with 5 ml buffer II (3.5% FCS, 0.5 M EDTA in HSS) at 37 °C for 15 min each time. Both suspensions of buffer II were combined and stored on ice. Colon samples were transferred to buffer III (10% FCS, 5 mg/ml DNAse, 125 U/mg Collagenase D in RPMI) and incubated for 60 min under the same conditions. After incubation, the suspension was vigorously shaken, and the remaining tissue was discarded. All cell suspensions of the colon were mixed and washed once with MACS buffer, and the cell subset composition was analyzed by flow cytometry using the following antibodies: CD3-APC-Cy7, CD8-PE-Cy7, CD11b-AF488, and MHCII-BV510 (all acquired from Biolegend, San Diego, USA); CD4-VioGreen™, B220-VioBlue^®^, and NK1.1-PerCP-Vio700 (all acquired from Miltenyi, Bergisch Gladbach, Germany); CD11c-APC (BD Biosciences, Heidelberg, Germany); and IgA-PE (Bio-Rad Laboratories, München, Germany). Flow cytometric analysis was performed using a flow cytometer (Gallios™, Beckmann Coulter, Brea, USA) and Kaluza Analysis 1.3 software (Beckmann Coulter, Brea, USA).

### Enzyme-linked immunosorbent assay (ELISA)

For protein isolation, MAT was homogenized in 1 mL extraction buffer (Takara Bio, Kusatsu, Japan) by a tissue homogenizer (Ultra Turrax, IKA^®^-Werke, Staufen, Germany) for 30 seconds. Afterwards, samples were incubated for 10 min on ice and subsequently centrifuged at 400 rpm for 10 min. A Bradford protein assay was performed to determine the protein concentrations in the obtained supernatants. A quantitative sandwich ELISA (Mouse Heme Oxygenase-1 EIA Kit, Takara Bio, Kusatsu, Japan) was used to determine the concentration of heme oxygenase 1 (HMOX1) in the obtained protein solutions from MAT. Initially, samples were diluted 1:1000, and the following procedure was performed according to the manufacturer’s instructions. Samples and standards were prepared in duplicate and measured at 450 nm with a plate reader (VICTOR™ X3, PerkinElmer, Waltham, MA, USA).

### Microbiome analysis

The intestinal cecum content was removed under sterile conditions. Then, DNA was extracted and the V1-V2 region of the 16 S rRNA gene was amplified and sequenced on the Illumina MiSeq platform as previously described^53^. Merging of paired-end raw reads were implemented according to Cole *et al*. and resulted in 32369 ± 26255 sequences per sample^54^. These sequences were subsequently assigned a taxonomic affiliation using RDP’s naive Bayesian classifier and rarefied to an equal depth (7398 sequences)^54^. Subsequent analyses were performed at the genus level. Calculations on diversity and non–metric multidimensional scaling analysis (metaMDS, auto transform = TRUE) were performed in R.

### Statistical analysis

All statistical analyses were performed using GraphPad Prism^®^6 software. Data were tested for normality with the D’Agostino-Pearson (n ≥ 8) normality test. For smaller sample sizes, the Shapiro-Wilk normality test (n ≥ 7) or Kolmogorov-Smirnov test (n ≥ 5) were used.

Quantitative two group parametric data were analyzed with a t test, whereas data from at least three groups were analyzed by one-way analysis of variance (ANOVA) with Tukey’s test for multiple comparisons. Nonparametric data for more than two groups were analyzed by the Kruskal-Wallis test with Dunn’s multiple comparisons test. Comparison of data with two factors was analyzed by using two-way ANOVA with Sidak’s multiple comparisons test. The significance level was set at 5%.

## Supplementary information

Supplementary information

## Data Availability

The datasets generated and analyzed in the current study are available from the corresponding author upon reasonable request.
