# Supplementary material for: A combination of genetics and microbiota influences the severity of the obesity phenotype in diet-induced obesity
Source: Sci Rep. 2020 Apr 9;10:6118. doi: 10.1038/s41598-020-63340-w (PMC7145845; doi:10.1038/s41598-020-63340-w)
Supplement: Supplementary file 1 — Supplementary information [file 41598_2020_63340_MOESM1_ESM.docx]

**Supplementary information**

**A combination of genetics and microbiota influences the severity of the obesity phenotype in diet-induced obesity**

Margarethe Smoczek^1^, Marius Vital^2^, Dirk Wedekind^1^, Marijana Basic^1^, Nils Holger Zschemisch^1^, Dietmar H. Pieper^2^, Anja Siebert^1^, Andre Bleich^1^, Manuela Buettner^1^

^1^ Institute of Laboratory Animal Science, Hannover Medical School, Hannover, Germany

^2^ Microbial Interactions and Processes (MINP), Helmholtz Centre for Infection Research (HZI), Braunschweig, Germany

**Supplemental Figures**

Wed Sep 05, 2018 9:36 CEST

C57BL/6J.ape from 1 to 3144

Alignment to

C57BL/6J HanZTm.ape-- Matches:3136; Mismatches:0; Gaps:8; Unattempted:0

C57BL/6N.ape-- Matches:3136; Mismatches:0; Gaps:8; Unattempted:0

C57BL/6J.ape-- Matches:3136; Mismatches:0; Gaps:8; Unattempted:0

* * * * * * * * * *

1>ggagagga**caggacaggaggagagtgtaaactagaggagggcgaaggaagcgaggcgcgacacagctcccggagaggagggcagaactgagcgcctagcg**>100

1>~~~~~~~~caggacaggaggagagtgtaaactagaggagggcgaaggaagcgaggcgcgacacagctcccggagaggagggcagaactgagcgcctagcg>92

1>~~~~~~~~caggacaggaggagagtgtaaactagaggagggcgaaggaagcgaggcgcgacacagctcccggagaggagggcagaactgagcgcctagcg>92

1>~~~~~~~~caggacaggaggagagtgtaaactagaggagggcgaaggaagcgaggcgcgacacagctcccggagaggagggcagaactgagcgcctagcg>92

* * * * * * * * * *

101>**cagagcggctcgcccggcctagaggagggagtgctgcgcagagaggaggggcttgaggccccgtagaaatgtcaatcagagcccggagcccagggaatct**>200

93>cagagcggctcgcccggcctagaggagggagtgctgcgcagagaggaggggcttgaggccccgtagaaatgtcaatcagagcccggagcccagggaatct>192

93>cagagcggctcgcccggcctagaggagggagtgctgcgcagagaggaggggcttgaggccccgtagaaatgtcaatcagagcccggagcccagggaatct>192

93>cagagcggctcgcccggcctagaggagggagtgctgcgcagagaggaggggcttgaggccccgtagaaatgtcaatcagagcccggagcccagggaatct>192

* * * * * * * * * *

201>**cggccaatctgttcggacctgacctgactcctcgctgccgccgctgccgccgccgccgcccgccgcggagcagatcaataggcgaacgcggagcgcgacg**>300

193>cggccaatctgttcggacctgacctgactcctcgctgccgccgctgccgccgccgccgcccgccgcggagcagatcaataggcgaacgcggagcgcgacg>292

193>cggccaatctgttcggacctgacctgactcctcgctgccgccgctgccgccgccgccgcccgccgcggagcagatcaataggcgaacgcggagcgcgacg>292

193>cggccaatctgttcggacctgacctgactcctcgctgccgccgctgccgccgccgccgcccgccgcggagcagatcaataggcgaacgcggagcgcgacg>292

* * * * * * * * * *

301>**cagcgcggcccgggctcggcccagcccccgatgcgctccggagccggggtccggagctgagcacggcggctcgggggtccgggcccccagtccgtaggtg**>400

293>cagcgcggcccgggctcggcccagcccccgatgcgctccggagccggggtccggagctgagcacggcggctcgggggtccgggcccccagtccgtaggtg>392

293>cagcgcggcccgggctcggcccagcccccgatgcgctccggagccggggtccggagctgagcacggcggctcgggggtccgggcccccagtccgtaggtg>392

293>cagcgcggcccgggctcggcccagcccccgatgcgctccggagccggggtccggagctgagcacggcggctcgggggtccgggcccccagtccgtaggtg>392

* * * * * * * * * *

401>**cgccgcgggccaccatgtccttcccccagctcggataccagtacatccgccctctctacccacccgaacgcccgggagctgcgggcggcggtggaggcgg**>500

393>cgccgcgggccaccatgtccttcccccagctcggataccagtacatccgccctctctacccacccgaacgcccgggagctgcgggcggcggtggaggcgg>492

393>cgccgcgggccaccatgtccttcccccagctcggataccagtacatccgccctctctacccacccgaacgcccgggagctgcgggcggcggtggaggcgg>492

393>cgccgcgggccaccatgtccttcccccagctcggataccagtacatccgccctctctacccacccgaacgcccgggagctgcgggcggcggtggaggcgg>492

* * * * * * * * * *

501>**cagtagcgctgggggccggagcggtcccggcgccggagcctccgagttggccgcctctgggtccctatccaatgtgctttcatcagtgtacggggcaccc**>600

493>cagtagcgctgggggccggagcggtcccggcgccggagcctccgagttggccgcctctgggtccctatccaatgtgctttcatcagtgtacggggcaccc>592

493>cagtagcgctgggggccggagcggtcccggcgccggagcctccgagttggccgcctctgggtccctatccaatgtgctttcatcagtgtacggggcaccc>592

493>cagtagcgctgggggccggagcggtcccggcgccggagcctccgagttggccgcctctgggtccctatccaatgtgctttcatcagtgtacggggcaccc>592

* * * * * * * * * *

601>**tacgccgctgccgcagctgccgccgccgccgctcagggttacggcgccttcctgccctacgccacggagctgcccatcttcccacagctggtaagagcca**>700

593>tacgccgctgccgcagctgccgccgccgccgctcagggttacggcgccttcctgccctacgccacggagctgcccatcttcccacagctggtaagagcca>692

593>tacgccgctgccgcagctgccgccgccgccgctcagggttacggcgccttcctgccctacgccacggagctgcccatcttcccacagctggtaagagcca>692

593>tacgccgctgccgcagctgccgccgccgccgctcagggttacggcgccttcctgccctacgccacggagctgcccatcttcccacagctggtaagagcca>692

* * * * * * * * * *

701>**gagaaacccgggagagaggggttggagttggggtgtggggcaaggtgagcgtgcagaagaaagctggggtcccggcgggaggataaaggtggggacggca**>800

693>gagaaacccgggagagaggggttggagttggggtgtggggcaaggtgagcgtgcagaagaaagctggggtcccggcgggaggataaaggtggggacggca>792

693>gagaaacccgggagagaggggttggagttggggtgtggggcaaggtgagcgtgcagaagaaagctggggtcccggcgggaggataaaggtggggacggca>792

693>gagaaacccgggagagaggggttggagttggggtgtggggcaaggtgagcgtgcagaagaaagctggggtcccggcgggaggataaaggtggggacggca>792

* * * * * * * * * *

801>**ggagctcactcgcgctccctgcgcgcgtgtccccttccttttccatgtgcgtacagggcgctcagtatgagctgaaggacagccctggcgtccagcatcc**>900

793>ggagctcactcgcgctccctgcgcgcgtgtccccttccttttccatgtgcgtacagggcgctcagtatgagctgaaggacagccctggcgtccagcatcc>892

793>ggagctcactcgcgctccctgcgcgcgtgtccccttccttttccatgtgcgtacagggcgctcagtatgagctgaaggacagccctggcgtccagcatcc>892

793>ggagctcactcgcgctccctgcgcgcgtgtccccttccttttccatgtgcgtacagggcgctcagtatgagctgaaggacagccctggcgtccagcatcc>892

* * * * * * * * * *

901>**ggccacggccgccgcgttcccgcacccgcaccccgccttctacccctatggccagtaccagttcggggacccgtcccgtcccaagaacgccacccgggaa**>1000

893>ggccacggccgccgcgttcccgcacccgcaccccgccttctacccctatggccagtaccagttcggggacccgtcccgtcccaagaacgccacccgggaa>992

893>ggccacggccgccgcgttcccgcacccgcaccccgccttctacccctatggccagtaccagttcggggacccgtcccgtcccaagaacgccacccgggaa>992

893>ggccacggccgccgcgttcccgcacccgcaccccgccttctacccctatggccagtaccagttcggggacccgtcccgtcccaagaacgccacccgggaa>992

* * * * * * * * * *

1001>**agcacgagcacgctcaaggcctggctcaacgagcaccgcaagaacccgtaccccaccaagggcgagaagatcatgttggccatcatcaccaagatgaccc**>1100

993>agcacgagcacgctcaaggcctggctcaacgagcaccgcaagaacccgtaccccaccaagggcgagaagatcatgttggccatcatcaccaagatgaccc>1092

993>agcacgagcacgctcaaggcctggctcaacgagcaccgcaagaacccgtaccccaccaagggcgagaagatcatgttggccatcatcaccaagatgaccc>1092

993>agcacgagcacgctcaaggcctggctcaacgagcaccgcaagaacccgtaccccaccaagggcgagaagatcatgttggccatcatcaccaagatgaccc>1092

* * * * * * * * * *

1101>**tcacccaggtgtccacctggttcgccaacgcgcgccggcgcctcaagaaggagaacaagatgacgtgggctccccgtagtcgcacggacgaggagggcaa**>1200

1093>tcacccaggtgtccacctggttcgccaacgcgcgccggcgcctcaagaaggagaacaagatgacgtgggctccccgtagtcgcacggacgaggagggcaa>1192

1093>tcacccaggtgtccacctggttcgccaacgcgcgccggcgcctcaagaaggagaacaagatgacgtgggctccccgtagtcgcacggacgaggagggcaa>1192

1093>tcacccaggtgtccacctggttcgccaacgcgcgccggcgcctcaagaaggagaacaagatgacgtgggctccccgtagtcgcacggacgaggagggcaa>1192

* * * * * * * * * *

1201>**tgcttatgggagcgagcgggaggaggaggacgaagaggaagacgaggaagagagcaaacgcgagctggagatggaggaggaggagctcgcaggagaggag**>1300

1193>tgcttatgggagcgagcgggaggaggaggacgaagaggaagacgaggaagagagcaaacgcgagctggagatggaggaggaggagctcgcaggagaggag>1292

1193>tgcttatgggagcgagcgggaggaggaggacgaagaggaagacgaggaagagagcaaacgcgagctggagatggaggaggaggagctcgcaggagaggag>1292

1193>tgcttatgggagcgagcgggaggaggaggacgaagaggaagacgaggaagagagcaaacgcgagctggagatggaggaggaggagctcgcaggagaggag>1292

* * * * * * * * * *

1301>**gaggacacggggggcgaggggctggccgacgacgacgaggatgaggagatcgatttggaaaacttagacagcgcggcagccgggtcggagctgaccctgg**>1400

1293>gaggacacggggggcgaggggctggccgacgacgacgaggatgaggagatcgatttggaaaacttagacagcgcggcagccgggtcggagctgaccctgg>1392

1293>gaggacacggggggcgaggggctggccgacgacgacgaggatgaggagatcgatttggaaaacttagacagcgcggcagccgggtcggagctgaccctgg>1392

1293>gaggacacggggggcgaggggctggccgacgacgacgaggatgaggagatcgatttggaaaacttagacagcgcggcagccgggtcggagctgaccctgg>1392

* * * * * * * * * *

1401>**ctggggcggcgcacaggaacggcgacttcggcctgggacccatttcggactgcaaaactagcgactcggacgacagctccgaaggcttggaggaccgacc**>1500

1393>ctggggcggcgcacaggaacggcgacttcggcctgggacccatttcggactgcaaaactagcgactcggacgacagctccgaaggcttggaggaccgacc>1492

1393>ctggggcggcgcacaggaacggcgacttcggcctgggacccatttcggactgcaaaactagcgactcggacgacagctccgaaggcttggaggaccgacc>1492

1393>ctggggcggcgcacaggaacggcgacttcggcctgggacccatttcggactgcaaaactagcgactcggacgacagctccgaaggcttggaggaccgacc>1492

* * * * * * * * * *

1501>**actgtcggtcctgagcctggccccaccgccaccgcccgtggccagggctcctgcatctccaccttctccaccctccagcctggatccctgcgctccggcc**>1600

1493>actgtcggtcctgagcctggccccaccgccaccgcccgtggccagggctcctgcatctccaccttctccaccctccagcctggatccctgcgctccggcc>1592

1493>actgtcggtcctgagcctggccccaccgccaccgcccgtggccagggctcctgcatctccaccttctccaccctccagcctggatccctgcgctccggcc>1592

1493>actgtcggtcctgagcctggccccaccgccaccgcccgtggccagggctcctgcatctccaccttctccaccctccagcctggatccctgcgctccggcc>1592

* * * * * * * * * *

1601>**ccggcgccctcctccgccctccagaagcccaagatctggtcactggccgagacggccaccagcccggacaacccacgccgctcgcctcccggagccggag**>1700

1593>ccggcgccctcctccgccctccagaagcccaagatctggtcactggccgagacggccaccagcccggacaacccacgccgctcgcctcccggagccggag>1692

1593>ccggcgccctcctccgccctccagaagcccaagatctggtcactggccgagacggccaccagcccggacaacccacgccgctcgcctcccggagccggag>1692

1593>ccggcgccctcctccgccctccagaagcccaagatctggtcactggccgagacggccaccagcccggacaacccacgccgctcgcctcccggagccggag>1692

* * * * * * * * * *

1701>**gttcgcctcccggcgcagccgttgcgcccccgacgctgcagctctcgcccgcggccgctgcagcggctgcagcggcacacagactcgtgtcggcgccgct**>1800

1693>gttcgcctcccggcgcagccgttgcgcccccgacgctgcagctctcgcccgcggccgctgcagcggctgcagcggcacacagactcgtgtcggcgccgct>1792

1693>gttcgcctcccggcgcagccgttgcgcccccgacgctgcagctctcgcccgcggccgctgcagcggctgcagcggcacacagactcgtgtcggcgccgct>1792

1693>gttcgcctcccggcgcagccgttgcgcccccgacgctgcagctctcgcccgcggccgctgcagcggctgcagcggcacacagactcgtgtcggcgccgct>1792

* * * * * * * * * *

1801>**tggcaaattccccgcttggaccaacaggcctttcccagggccgccggccggtccgcggccgcacccgctgtccatgctgggctcggccccacagcacctg**>1900

1793>tggcaaattccccgcttggaccaacaggcctttcccagggccgccggccggtccgcggccgcacccgctgtccatgctgggctcggccccacagcacctg>1892

1793>tggcaaattccccgcttggaccaacaggcctttcccagggccgccggccggtccgcggccgcacccgctgtccatgctgggctcggccccacagcacctg>1892

1793>tggcaaattccccgcttggaccaacaggcctttcccagggccgccggccggtccgcggccgcacccgctgtccatgctgggctcggccccacagcacctg>1892

* * * * * * * * * *

1901>**ctgggacttcccggagccgcaggtcatcccgctgcggccgcagccgcctatgctcggcctgccgagccggagagtggaacaggtgacaagaaaaatgtag**>2000

1893>ctgggacttcccggagccgcaggtcatcccgctgcggccgcagccgcctatgctcggcctgccgagccggagagtggaacaggtgacaagaaaaatgtag>1992

1893>ctgggacttcccggagccgcaggtcatcccgctgcggccgcagccgcctatgctcggcctgccgagccggagagtggaacaggtgacaagaaaaatgtag>1992

1893>ctgggacttcccggagccgcaggtcatcccgctgcggccgcagccgcctatgctcggcctgccgagccggagagtggaacaggtgacaagaaaaatgtag>1992

* * * * * * * * * *

2001>**gggcacagtgggagggtgttggtggggctctcgcctagactaaagggaaagaacggggctttgggggtgtgtgggacgccggtgttttcctctttgctcg**>2100

1993>gggcacagtgggagggtgttggtggggctctcgcctagactaaagggaaagaacggggctttgggggtgtgtgggacgccggtgttttcctctttgctcg>2092

1993>gggcacagtgggagggtgttggtggggctctcgcctagactaaagggaaagaacggggctttgggggtgtgtgggacgccggtgttttcctctttgctcg>2092

1993>gggcacagtgggagggtgttggtggggctctcgcctagactaaagggaaagaacggggctttgggggtgtgtgggacgccggtgttttcctctttgctcg>2092

* * * * * * * * * *

2101>**caggcgggtgcagctgccctggaggcctggagcctgtgttttaaaggcctcctttttcttccctctgtttcctacagatcgctgtagtgccttggaagtg**>2200

2093>caggcgggtgcagctgccctggaggcctggagcctgtgttttaaaggcctcctttttcttccctctgtttcctacagatcgctgtagtgccttggaagtg>2192

2093>caggcgggtgcagctgccctggaggcctggagcctgtgttttaaaggcctcctttttcttccctctgtttcctacagatcgctgtagtgccttggaagtg>2192

2093>caggcgggtgcagctgccctggaggcctggagcctgtgttttaaaggcctcctttttcttccctctgtttcctacagatcgctgtagtgccttggaagtg>2192

* * * * * * * * * *

2201>**gagaaaaagttactcaagacagctttccagccggtgccaaggcggtaagagactccgaatttgggaagcatagtgggagacccttcgggaggaataagaa**>2300

2193>gagaaaaagttactcaagacagctttccagccggtgccaaggcggtaagagactccgaatttgggaagcatagtgggagacccttcgggaggaataagaa>2292

2193>gagaaaaagttactcaagacagctttccagccggtgccaaggcggtaagagactccgaatttgggaagcatagtgggagacccttcgggaggaataagaa>2292

2193>gagaaaaagttactcaagacagctttccagccggtgccaaggcggtaagagactccgaatttgggaagcatagtgggagacccttcgggaggaataagaa>2292

* * * * * * * * * *

2301>**aaacacagagccagtggcgggagccctttgccccctgggaaggtagcaaagtagctggcaaactattgctaaaggacccaaaaaatccactcttctgtca**>2400

2293>aaacacagagccagtggcgggagccctttgccccctgggaaggtagcaaagtagctggcaaactattgctaaaggacccaaaaaatccactcttctgtca>2392

2293>aaacacagagccagtggcgggagccctttgccccctgggaaggtagcaaagtagctggcaaactattgctaaaggacccaaaaaatccactcttctgtca>2392

2293>aaacacagagccagtggcgggagccctttgccccctgggaaggtagcaaagtagctggcaaactattgctaaaggacccaaaaaatccactcttctgtca>2392

* * * * * * * * * *

2401>**cttcttgtggacccatagacacggttccattctcggctggagcacctcaggcctgtggtacagagctaggggaagtctggcccttacaagaggcctgtgg**>2500

2393>cttcttgtggacccatagacacggttccattctcggctggagcacctcaggcctgtggtacagagctaggggaagtctggcccttacaagaggcctgtgg>2492

2393>cttcttgtggacccatagacacggttccattctcggctggagcacctcaggcctgtggtacagagctaggggaagtctggcccttacaagaggcctgtgg>2492

2393>cttcttgtggacccatagacacggttccattctcggctggagcacctcaggcctgtggtacagagctaggggaagtctggcccttacaagaggcctgtgg>2492

* * * * * * * * * *

2501>**cgcagaaggcctctgcgtaatgtccacttccttccaagcccggtcttgacttctgatctctttagctcagcttttagttaatactcccacagctaaacac**>2600

2493>cgcagaaggcctctgcgtaatgtccacttccttccaagcccggtcttgacttctgatctctttagctcagcttttagttaatactcccacagctaaacac>2592

2493>cgcagaaggcctctgcgtaatgtccacttccttccaagcccggtcttgacttctgatctctttagctcagcttttagttaatactcccacagctaaacac>2592

2493>cgcagaaggcctctgcgtaatgtccacttccttccaagcccggtcttgacttctgatctctttagctcagcttttagttaatactcccacagctaaacac>2592

* * * * * * * * * *

2601>**cggctttataggggatcccctatctaagcagtctccccaactctaaagctcactgtctctgcttccgttctcttccaggccacagaaccatctggacgct**>2700

2593>cggctttataggggatcccctatctaagcagtctccccaactctaaagctcactgtctctgcttccgttctcttccaggccacagaaccatctggacgct>2692

2593>cggctttataggggatcccctatctaagcagtctccccaactctaaagctcactgtctctgcttccgttctcttccaggccacagaaccatctggacgct>2692

2593>cggctttataggggatcccctatctaagcagtctccccaactctaaagctcactgtctctgcttccgttctcttccaggccacagaaccatctggacgct>2692

* * * * * * * * * *

2701>**gctctggtcttatcagctctctcctcgtcttaacttttcaaccatttttaaatcgttgtactaattgtaaaaaacaaaacaaaacaaaatcgctctgtat**>2800

2693>gctctggtcttatcagctctctcctcgtcttaacttttcaaccatttttaaatcgttgtactaattgtaaaaaacaaaacaaaacaaaatcgctctgtat>2792

2693>gctctggtcttatcagctctctcctcgtcttaacttttcaaccatttttaaatcgttgtactaattgtaaaaaacaaaacaaaacaaaatcgctctgtat>2792

2693>gctctggtcttatcagctctctcctcgtcttaacttttcaaccatttttaaatcgttgtactaattgtaaaaaacaaaacaaaacaaaatcgctctgtat>2792

* * * * * * * * * *

2801>**agtgacgagttgcaagcatgtctgtgtatgattaccttaaaagaaacactaaacaacaacaacaacaacaacaacaacaggaaaggacaaaagaaagaaa**>2900

2793>agtgacgagttgcaagcatgtctgtgtatgattaccttaaaagaaacactaaacaacaacaacaacaacaacaacaacaggaaaggacaaaagaaagaaa>2892

2793>agtgacgagttgcaagcatgtctgtgtatgattaccttaaaagaaacactaaacaacaacaacaacaacaacaacaacaggaaaggacaaaagaaagaaa>2892

2793>agtgacgagttgcaagcatgtctgtgtatgattaccttaaaagaaacactaaacaacaacaacaacaacaacaacaacaggaaaggacaaaagaaagaaa>2892

* * * * * * * * * *

2901>**gaaagccagtccccccaccttggccctcccccaagtcaccccacatttagattgtgaggttccttcgttgtgttgatttcggggggggggggagtggggt**>3000

2893>gaaagccagtccccccaccttggccctcccccaagtcaccccacatttagattgtgaggttccttcgttgtgttgatttcggggggggggggagtggggt>2992

2893>gaaagccagtccccccaccttggccctcccccaagtcaccccacatttagattgtgaggttccttcgttgtgttgatttcggggggggggggagtggggt>2992

2893>gaaagccagtccccccaccttggccctcccccaagtcaccccacatttagattgtgaggttccttcgttgtgttgatttcggggggggggggagtggggt>2992

* * * * * * * * * *

3001>**tacggagagaacaagcctgtctgactctttttgtatatacgggaaaatgtacatacgtgtgaaccaaattgtacaagaaagtatctattttttggctaaa**>3100

2993>tacggagagaacaagcctgtctgactctttttgtatatacgggaaaatgtacatacgtgtgaaccaaattgtacaagaaagtatctattttttggctaaa>3092

2993>tacggagagaacaagcctgtctgactctttttgtatatacgggaaaatgtacatacgtgtgaaccaaattgtacaagaaagtatctattttttggctaaa>3092

2993>tacggagagaacaagcctgtctgactctttttgtatatacgggaaaatgtacatacgtgtgaaccaaattgtacaagaaagtatctattttttggctaaa>3092

* * * *

3101>**taaatgagctgttgccacttttgactataaccggcaggtctgcc**>3144

3093>taaatgagctgttgccacttttgactataaccggcaggtctgcc>3136

3093>taaatgagctgttgccacttttgactataaccggcaggtctgcc>3136

3093>taaatgagctgttgccacttttgactataaccggcaggtctgcc>3136
